# Reduced neonatal brain-derived neurotrophic factor is associated with autism spectrum disorders

**DOI:** 10.1038/s41398-019-0587-2

**Published:** 2019-10-07

**Authors:** Kristin Skogstrand, Christian Munch Hagen, Nis Borbye-Lorenzen, Michael Christiansen, Jonas Bybjerg-Grauholm, Marie Bækvad-Hansen, Thomas Werge, Anders Børglum, Ole Mors, Merethe Nordentoft, Preben Bo Mortensen, David Michael Hougaard

**Affiliations:** 1 Danish Center for Neonatal Screening, Department for Congenital Disorders, Statens Serum Institut, Copenhagen, Denmark; 2 iPSYCH, The Lundbeck Foundation Initiative for Integrative Psychiatric Research, Copenhagen, Denmark; 3 Department of Biomedical Science, University of Copenhagen, Copenhagen, Denmark; 4 Institute of Biological Psychiatry, Mental Health Centre Sct. Hans, Copenhagen University Hospital, Copenhagen, Denmark; 5 Department of Clinical Medicine, Faculty of Health and Medical Sciences, University of Copenhagen, Copenhagen, Denmark; 6 Department of Biomedicine and iSEQ, Centre for Integrative Sequencing, Aarhus University, Aarhus, Denmark; 7 National Centre for Register-Based Research, Business and Social Sciences, Aarhus University, Aarhus, Denmark; 8 Center for Genomics and Personalized Medicine, Aarhus, Denmark; 9 Psychosis Research Unit, Aarhus University Hospital—Psychiatry, Aarhus, Denmark; 10 Department of Clinical Medicine, Aarhus University Hospital, Aarhus, Denmark; 11 Mental Health Centre Copenhagen, Copenhagen University Hospital, Copenhagen, Denmark; 12 Centre for Integrated Register-based Research, CIRRAU, Aarhus University, Aarhus, Denmark

**Keywords:** Predictive markers, Molecular neuroscience

## Abstract

Mental disorders have for the majority of cases an unknown etiology, but several studies indicate that neurodevelopmental changes happen in utero or early after birth. We performed a nested case–control study of the relation between blood levels of neuro-developmental (S100B, BDNF, and VEGF-A) and inflammatory (MCP-1, TARC, IL-8, IL-18, CRP, and IgA) biomarkers in newborns, and later development of autism spectrum disorders (ASD, *N* = 751), attention deficit hyperactivity disorders (ADHD, *N* = 801), schizophrenia (*N* = 1969), affective (*N* = 641) or bipolar disorders (*N* = 641). Samples and controls were obtained as part of the iPSYCH Danish Case–Cohort Study using dried blood spot samples collected between 1981 and 2004, and stored frozen at the Danish National Biobank. In newborns lower blood level of BDNF was significantly associated with increased odds (OR 1.15) of developing ASD (*p* = 0.001). This difference could not be explained by genetic variation in the BDNF coding gene region. A tendency of decreased levels of all the neurotrophic markers and increased levels of all inflammatory markers was noted. The low newborn blood levels of BDNF in children developing ASD is an important finding, suggesting that lower BDNF levels in newborns contributes to the etiology of ASD and indicates new directions for further research. It may also help identifying a long-sought marker for high-ASD risk in, e.g., younger siblings of ASD children.

## Introduction

Autism spectrum disorders (ASD), attention deficit hyperactivity disorder (ADHD), schizophrenia, affective- and bipolar disorders are all mental disorders with, for most cases, a largely unknown etiology^1–5^.

ASD and ADHD are normally diagnosed in childhood. ASD is a group of developmental disorders with qualitative abnormalities in reciprocal social interaction, communication and restricted, repetitive, and stereotyped patterns of behavior, interests, and activities. The symptoms of the disorders exhibit a high degree of variation, and their severity varies considerably^6^. ADHD is the most frequent mental diagnosis among children and is characterized by hyperactivity, impulsivity, and inattention^7^.

Schizophrenia and affective disorders are mainly diagnosed in youth and adulthood. Schizophrenia is a syndrome of symptoms characterized by psychosis, social withdrawal, and difficulties with social interactions^8^, and is generally accepted as not being a single disorder^5^. Affective disorders are a set of mood (affective) disorders^9^, where bipolar disorder is a sub group characterized by repeated episodes in which mood and activity levels are significantly disturbed by an elevation of mood, increased energy and activity or of a lowering of mood and decreased energy and activity^10^.

The patients often have more than one of the diagnoses, and the symptoms—as well as genetic architecture—overlap^11–15^. A recent study using structural magnetic resonance imaging of the brain in children has shown similar patterns between ASD and ADHD^16^. The mean life-time risk for the investigated disorders are 2.0% for ASD, 3.2% for ADHD, 1.8% for schizophrenia, 13.2% for affective disorders, and 1.6% for bipolar disorders^17^. ASD, schizophrenia, and ADHD are more often seen in monozygotic than in dizygotic twins, indicating strong genetic effects, and are more prevalent in males^18–20^. The heterogeneity of the disorders makes it difficult to elucidate their pathogenesis, which most probably is multi etiological^2–5^. Several causes of these disorders have been suggested: prenatal and early life stress^21^, brain synaptic dysfunctions^22^, early life exposure to metals^23^, perinatal hypoxia^24^, preterm birth^25^, neuro-inflammation^26^, neonatal infection^27^, maternal viral-infections^28^, maternal immune activation^29^, gut–microbe–brain–immune axis^30^, and genetic factors^31^ are all considered plausible explanations. Meta-analyses indicate that a combination of environmental and genetic factors, as well as both perinatal and neonatal factors, increase the risk^3,32,33^. The mechanisms of the neurodevelopmental defects are unknown, but a change in neuron synapse formation (i.e., a change in connections between brain areas) may be a part of the explanation^29^. There has been considerable research in the area, but no clinically relevant biomarker or specific risk factor has yet been identified^34–37^.

As inflammation is involved in several of the suggested causes for mental disorders, our aim in the current study was to explore the possible role that inflammation/infection may play in the developing brain of newborns. Further, we wanted to explore if different neurotrophic factors in newborns may contribute to the understanding of the etiology of mental disorders. The Danish Neonatal Screening Biobank makes it possible for us to analyze the dried blood spot samples (DBSS) drawn from all children 5–7 days after birth for neonatal screening^38^. We have previously developed multiplex analytical methods for these samples^39,40^ and have demonstrated that it is possible to measure a number of biomarkers in DBSS stored at −24 °C for more than 30 years^41–43^. As part of the Lundbeck Foundation Initiative for Integrative Psychiatric Research (iPSYCH)^44^, we selected cases and controls matched by birth year from the iPSYCH Danish Case–Cohort Study from newborns, where cases were diagnosed with ASD (801 cases and 2421 controls), ADHD (751 cases and 2423 controls), schizophrenia (1969 cases and 2681 controls), bipolar- (641 cases and 2311 controls), and affective (641 cases and 2371 controls) disorders. Nine biomarkers were selected, based on earlier described studies either as a promising biomarker for one or more of the disorders, or as a marker that indicate either brain damage or inflammation^34^. The following markers were selected to explore neuronal development: S100 calcium binding protein B (S100B), brain-derived neurotrophic factor (BDNF), and vascular endothelial growth factor A (VEGF-A), and to indicate neonatal inflammation/infection: monocyte chemotactic protein-1 (MCP-1), thymus and activation-regulated chemokine (TARC), interleukin-8 (IL-8), IL-18, C-reactive protein (CRP), and immunoglobulin A (IgA).

To further explore significant findings, we examined the genetic influence on the measured biomarker levels using SNPs from GWAS imputed data in the coding region of the BDNF gene. We also tested for association between genetic clusters, generated using principal component analysis and K-means clustering, and BDNF blood levels. In addition, correlation between BDNF and the other measured biomarkers were investigated.

## Methods

### Sample selection

Samples were selected from the iPSYCH cohort of 86 189 individuals^44^. Table 1 shows the ICD-10 codes for the cases, and the numbers, distribution and demographic data for cases and controls for each disorder. Most of the controls were used as controls for several case groups, and some of the cases have multiple diagnosis and thus are cases in more than one group. Controls were chosen at random from the entire Danish population and were matched with cases on birth year. In order to control for population stratification and cryptic relatedness effects on marker levels, which refers to the idea that some members of a case–control sample might be close relatives, in which case their genotypes are not independent draws from the population frequencies^45^, first- and second-degree relatives were removed, please see “Statistics” for details. The distribution of diagnoses in cases and controls in each group are summarized in Table 2. For more information on sample selection, please see Pedersen et al.^44^. The project was accepted by the Danish ethical committee (approval number 1-10-72-287-12). According to Danish law, all blood samples can legally be stored without explicit informed consent. Their later use for additional analysis are however conditional on approvals of each separate project from the Research Ethics Committee and the Danish Data Protection Agency. According to the law, the use of samples for research projects require informed consent, but the Research Ethics Committee can waive this requirement if the project is carried out with anonymous samples and does not in any way imply health-related risks or may burden the participants^46^.

**Table 1:** Demographic data for cases and controls

	Cases	Controls
*Number in each group*		
Schizophrenia (ICD-10 F20)	1969	2681
Bipolar disorders (ICD-10 F30–31)	641	2311
Affective disorders (ICD-10 F32–39)	641	2371
ASD (ICD-10 F84.0, −1, −5, −8, −9)	801	2421
ADHD (ICD-10 F90.0)	751	2423
*Gender distribution %male/%female*		
Schizophrenia	57/43	51/49
Bipolar disorders	40/60	51/49
Affective disorders	32/68	50/50
ASD	78/22	52/48
ADHD	73/27	52/48
*Birth weight (median [IQR] in gram)*		
Schizophrenia	3450 [3105–3805]	3505 [3155–3840]
Bipolar disorders	3425 [3105–3762]	3500 [3155–3810]
Affective disorders	3428 [3089–3755]	3500 [3155–3805]
ASD	3520 [3159–3900]	3505 [3175–3866]
ADHD	3502 [3151–3859]	3505 [3175–3865]
*Gestational age (median [IQR] in weeks)*		
Schizophrenia	40 [39–41]	40 [39–41]
Bipolar disorders	40 [39–41]	40 [39–41]
Affective disorders	40 [39–41]	40 [39–41]
ASD	40 [39–41]	40 [39–41]
ADHD	40 [39–41]	40 [39–41]
*Gestational age (week range,% of total)*	25–33 34–37 38–44	25–33 34–37 38–44
Schizophrenia	1,6 7,2 91,2	0,8 6,7 92,5
Bipolar disorders	1,9 6,8 91,3	0,9 6,5 92,7
Affective disorders	1,9 5,5 92,6	0,6 6,5 92,7
ASD	0,9 8,3 90,8	1,0 6,7 92,3
ADHD	1,2 9,3 89,5	1,0 6,7 92,3
*Age at diagnosis (median [IQR] in years)*		
Schizophrenia	21 [18–23]	
Bipolar disorders	22 [19–25]	
Affective disorders	19 [17–23]	
ASD	10 [7–13]	
ADHD	10 [8–15]	

Demographic data for cases and controls divided into the different disorders

**Table 2:** Distribution of diagnosis in cases and controls

Case diagnosis →	Schizophrenia		Bipolar disorders		Affective disorders		ASD		ADHD	
Add. diagnosis ↓	Cases	Controls	Cases	Controls	Cases	Controls	Cases	Controls	Cases	Controls
Schizophrenia	100	0	4.4	0.5	4.7	0.5	1.0	0.4	0.9	0.4
Bipolar disorders	3.0	0.3	100	0.1	0	0.3	0.1	0.2	0.5	0.2
Affective disorders	33.4	2.5			100	2.7	7.4	1.9	7.5	1.9
ASD	6.6	0.8	5.3	0.7	4.4	0.7	100	1.2	19.0	1.3
ADHD	8.3	0.8	12.0	0.7	6.7	0.8	21.8	0.9	100	0.9
Only case diagnosis	57.3	0.0	80.3	0.1	85.5	2.2	71.2	0.9	74.0	0.7

The numbers are shown as percentage of cases and controls of each diagnostic group shown in the top row that also have the diagnosis in the left column

### Sample analysis, proteins

Two 3.2 mm disks from the DBSS were punched into each well of polymerase chain reaction plates (Sarstedt, 72.1981.202). 130 µl extraction buffer (phosphate-buffered saline containing 1% bovine serum albumin (Sigma Aldrich #A4503), 0.5% Tween-20 (Merck Millipore #8.22184.0500), and complete protease inhibitor cocktail (Roche Diagnostics #11836145001)) were added to each well, and the samples were incubated for 1 h at room temperature on a microwell shaker set at (900 rpm). The extracts were manually moved to sterile Matrix 2D tubes (Thermo Scientific, 3232) and stored frozen at −80 °C. About 3 years later, samples were thawed and analyzed using Meso-Scale plates printed and customized for the project. Antibodies printed to the plates were specific for BDNF, CRP, IL-18, IL-8, IgA, MCP-1, S100B, TARC, and VEGF-A. All reagents, except for the reagents for S100B, were purchased from MesoScale Diagnostics (MSD), Maryland, US. The S100B assay was set up in-house before custom printing using capture antibody (Sigma-Aldrich, #S2532) and detection antibody (DAKO, #Z0311). Calibrators were custom made by MSD. The plates and calibrators were made in one batch to minimize assay variations. Controls were made in-house from part of the calibrator solution in one batch, aliquoted in portions for each plate and stored at −20 °C until use. The samples were prepared on the plates as recommended from the manufacturer, and were immediately read on the QuickPlex SQ 120 (MSD, MA, USA). Analyte concentrations were calculated from the calibrator curves on each plate using 4PL logistic regression using the MSD Workbench software.

### Sample analysis, genotyping

Array genotyping of all samples was made at the Broad Institute (Boston, MA, USA) using Infinium PsychChip v1.0 array (Illumina, San Diego, CA, USA), as previously described^44^.

### Analytical characterization

Intra-assay variations were calculated from 40 measurements of a pool of the same control sample on the same plate. Inter-assay variations were calculated from controls analyzed on each plate during the sample analysis, 131 plates in total. Limit of detections were calculated as 2.5 standard deviations from repeated measurements of the zero calibrator. The higher detection limits were defined as the highest calibrator concentrations.

### Preanalytical variation

To validate the stability of the samples during storage, we randomly selected 16 samples stored in the biobank since 1982, since 1990, since 2000, since 2010, and 16 fresh samples only 3–4 days old (from 2016). After extracting the samples and adding them to the MSD-plates, the rest of the extracts were frozen for 2 days, thawed and measured as described above to imitate the freeze–thaw cycle of the samples in the study.

### Statistics

Multiple logistic regression assessment of association between marker levels and diagnosis/phenotype: the measured concentrations for each marker were log-transformed (ln) and standardized by year using *z*-scores, in order to accommodate possible changes across the period. Each protein was analyzed on a continuous scale. First and second degree-relatives were removed (relatedness coefficient >0.2) and principal component analysis were used to adjust for population stratification. The relatedness coefficient were calculated using KING(v.1.9)^47^ on Infinium PsychChip v1.0 array (Illumina, San Diego, CA, USA) data after removal of INDELs and SNPs within long-range disequilibrium regions^48^ or with a missing-rate above 0.001. Population structure was inferred using principal components analysis using EIGENSOFT version 6.1.4^49^ smartPCA on the complete iPSYCH array dataset and outliers defined using default settings. Because the distribution of gender between cases and controls were not equal and due to gender-specific expression of some markers, we adjusted for gender in the statistical analysis. We also initially adjusted for gestational age and age in days of the newborns when the samples were drawn. The last two factors did not make any difference in the statistical analysis, and the results presented are thus only adjusted for gender. To be sure that gestational age did not have any influence on the BDNF results, we made the same calculations on only the term and premature cases and controls, respectively. The statistical analysis was done with logistic regression using R 3.3.1^50^. Multiple testing correction was done using Bonferroni correction at an α level of 0.05. A *p* value was considered statistically significant if *p* < 0.0056 (0.05/9 analytes).

Assessment of genetic influence on phenotype associated marker levels using multiple linear regression: to investigate whether genetic variation influences the marker levels we extracted genetic regions of interest, defined as a region containing all known NCBI refSeq^51^ transcript variants and GTEx eQTL (tissue = brain), from imputed data^15^. Genetic variants with call-percent <96% and imputation score ⩽0.9 and highly correlated genetic variants were removed based on the mean absolute correlation with a cutoff >0.75 using Caret package^52^. A multiple linear regression model was applied for all possible combinations of genetic variants, including gender in all models. Model selection was based on a minimum criteria of *p* < 0.01 for all independent variables and a minimum AIC and maximum adjusted R-squared.

Assessment of genetic clusters and association to marker levels and phenotype based on principal component- and clustering analysis: to assess whether genetic variation in regions of interest is associated with marker level or phenotype “risk” we used principal components analysis (R stats 3.5.1) to represent the most genetic variability in two-dimensions and cluster the result using K means clustering. The optimal *k* was defined by pre-analysis of total within sum of square minimization (R factoextra 1.0.5). Only complete observations were included in the analysis. The assessment of the association between clusters and marker levels was done using multiple linear regression modeling and the association between clusters and phenotype using multiple logistic regression.

The amount of degradation of the different analytes was calculated with a Mann Whitney nonparametric test using GraphPad Prism 7.03 (GraphPad Software, Inc., La Jolla, CA, USA).

The samples were barcoded in the neonatal screening where we did not have any knowledge of the children. These barcodes were used during sample analysis and cleaning of data, thus the investigators did not have any knowledge about case and control status.

## Results

**Fig. 1:**
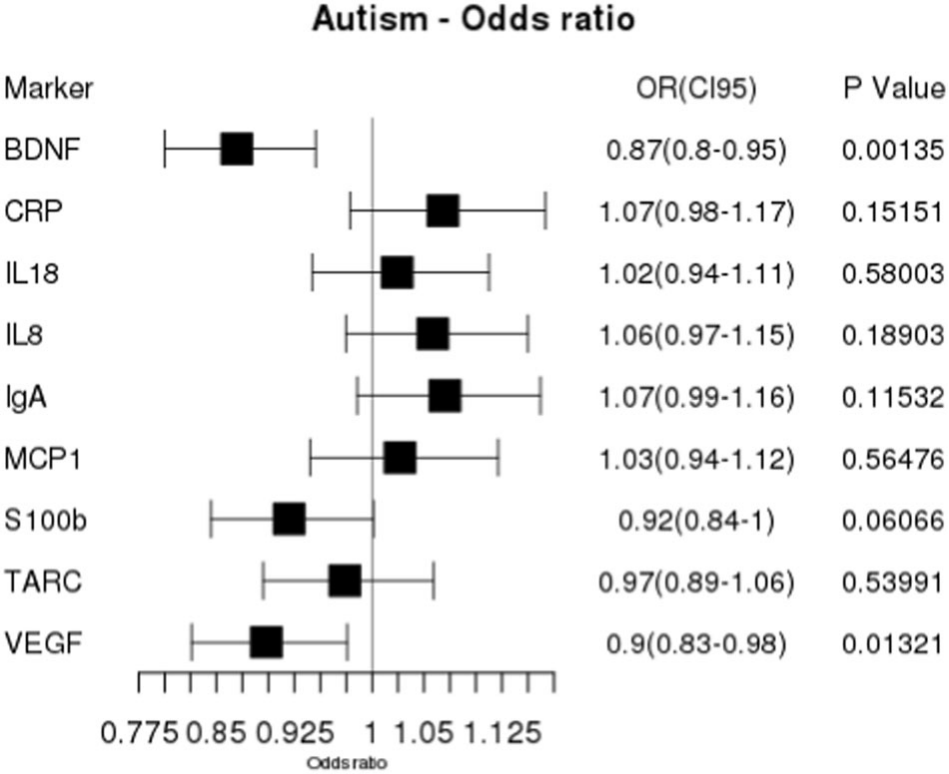
Forest plot for ASD. The figure shows calculated odds-ratios (OR with 95% confidence intervals) and *p* values for each marker. There was decreased OR for developing ASD with increasing level of BDNF. OR for developing ASD with low BDNF was 1/0.87 (1/0.95–1/0.8) = 1.15 (1.05–1.25)

**Fig. 2:**
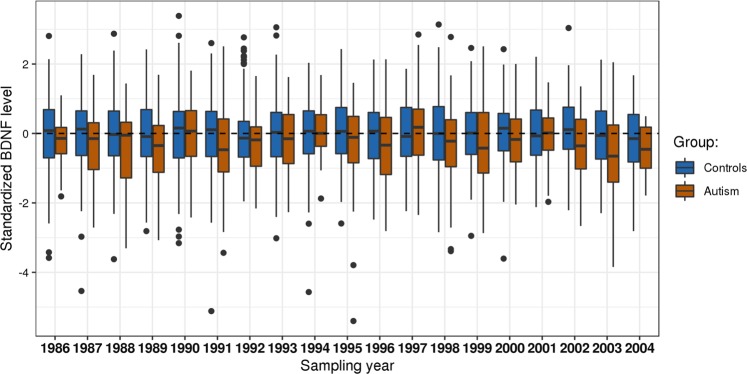
BDNF Z-scores for ASD cases and controls. The concentrations were standardized by year in cases (orange boxes) and controls (blue boxes) using *z*-scores. The figure shows median BDNF *z*-scores with 25–75th percentile (box), 1.5 × (25–75th) (vertical lines) and upper and lower values (dots)

Newborns later diagnosed with ASD had significantly decreased levels of the neurodevelopmental factor BDNF. In addition, there was a tendency toward decreased concentrations of all neurodevelopmental factors and increased concentrations of inflammatory markers, but the statistical significance for the other biomarkers disappeared after Bonferroni corrections (Fig. 1). Figure 2 shows the calculated *z*-scores for BDNF for ASD-cases and controls per year. Dividing the samples in only the term and premature cases and controls, respectively, to be sure that gestational age did not have any influence on the results, did not change the results (results not shown).

When performing pairwise correlation for the ASD cases, BDNF had a significant positive correlation with the other neurodevelopmental factors, VEGF and S100b, and with the inflammatory/infection markers IgA and TARC. The correlation with CRP was negative (Supplementary Fig. 1). There were no differences in correlation between females and males, nor between cases and controls, respectively (data not shown). There was no correlation between age at ASD diagnosis and BDNF level (data not shown).

A total of 122 genetic variants SNPs in the BDNF region (GRCh37:chr11: :27674440–27745605) was extracted, and after filtering on imputation score and call-percent, 98 remained and were reduced to 12 after removing correlated (>0.75) variants. The best fit model, according to described criteria (*R*^2^ = 0.042, *F*(2/2386) = 53.62, *p* < 2.2e−16) included refSNP ID:rs142615576 (T/C) (*β* = 0.27, *p* = 5.93e–5). However, rs142615576 explained only 0.75% of the variance and did not significantly contribute to the predictive ability of ASD in the logistic model (rs142615576, *p* = 0.40). The cumulative proportion of BDNF variance captured by principal component 1 and 2 was 89%, and the optimal *k* was estimated to 6 for the 98 variants included in the analysis. There was no significant correlation between the BDNF levels and any of the 6 clusters after correcting for multiple testing. Nor had the clusters a predictive value for ASD in a logistic regression model (data not shown).

We did not find any significant correlations between ADHD, schizophrenia, bipolar-, and affective disorders and the different biomarkers after Bonferroni corrections (Supplementary Figs. 2–5). The measured concentrations without any adjustments of all biomarkers for the different disorders are shown as box-plots in Supplementary Fig. 6.

### Analytical performance and sample stability

Median intra assay variation was 3.3% and median inter assay variation was 13.3%. Supplementary Table 1 shows assay variations and limit of detections for all analytes, and mean concentrations measured for all samples.

After being freezed and thawed once after extraction, we found the analytes to be relatively stable. BDNF, IL-18, S100B, and VEGF-A were significantly decreased overall if the extracts were freeze-thawed once, but mainly in the oldest samples. Generally, the analytes seem to be more fragile to a freeze–thaw cycle after more than 15 of years’ storage before the extraction. Overall, there was considerable degradation of the analytes over the years, but all analytes were measurable in the samples even after 30 years of storage (Supplementary Table 2).

## Discussion

This is to our knowledge the largest study ever reported regarding biomarkers in newborns later diagnosed with mental disorders. The most significant finding was the association between later diagnosis of ASD and decreased neonatal blood levels of BDNF.

BDNF is the main neurotrophic cytokine in the central nervous system. It contributes to pre- and postnatal brain development^53^; by promoting neuronal growth, differentiation, and survival^54^, and by its potent effects on synaptic and structural plasticity^55^. BDNF is present both at pre- and postsynaptic sites, facilitating the release of neurotransmitters, and promoting the function of ion-transmitters and NMDA receptors^56^. Overall, BDNF appears to strengthen excitatory (glutamatergic) synapses and weaken inhibitory (GABAergic) synapses^54^. Outside the brain, megakaryocytes (cells that produce blood platelets), salivary glands, and endothelial cells produce BDNF^57^. Blood platelets store most of the blood BDNF and release BDNF upon activation at the site of traumatic injury to facilitate the repair of peripheral nerves or other tissues where it binds to tropomyosin-related kinase receptor type B (TrkB) with high affinity, and pan neurotrophin receptor of p75NTR with low affinity^58^. BDNF is secreted from secretory vesicles, partly constitutively but mainly stimulation- and activity-dependent^59^. Animal studies have shown that BDNF can cross the blood brain barrier both ways^60^. Thus, concentrations measured in the blood are most probably correlated with concentrations in the brain.

The decreased levels of BDNF that we found in DBSS from newborns who later develop ASD concur with the findings in a smaller study (414 ASD, 820 controls) on DBSS from the Danish Neonatal Screening Biobank^61^, although there may be some overlap of the cases between the two studies. An earlier small study on DBSS in newborns has shown increased levels of BDNF compared to controls^62^. This was a small study (60 ASD and 54 controls) based on single antibody detection, and the authors have not been able to reproduce their results using another analytical method^63^. Another small study (84 ASD and 159 controls) on DBSS from newborns found no difference in BDNF levels in ASD cases compared to controls^64^. The DBSS was drawn from newborns 1–2 days after birth compared to 5–7 days in our study. Thus, both study size and sampling time can probably explain the different results. A study on ASD children age 0–19 years old has shown that ASD cases age 0–9 years old have lower serum BDNF-levels compared to controls, whereas the age 10–19 year old ASD cases have higher levels than the controls^65^. A meta-analysis of 20 studies analyzing blood BDNF levels in children that have already been diagnosed with ASD (887 cases, 901 controls) reported higher levels of BDNF in ASD cases than controls^56^. Thus, the BDNF expression seems to be delayed in the ASD cases. When comparing results from different immunoassays, we have to be aware that antibodies in various assays may capture different parts of the BDNF molecule, thus this may also explain differences seen when comparing studies. A study has described elevated numbers of blood platelets in ASD cases (mean age 11.9 years)^66^, but we were not able to find similar studies in newborns. As blood platelets store BDNF^58^, this would be relevant to explore in further studies. We do not know if we are measuring the total BDNF in dried blood spot samples, that is, if the platelet-bound BDNF is released, or if we are only measuring the free BDNF. To further elucidate the role of BDNF in the development of ASD it would be relevant to investigate the expression levels of TrkB, the receptor that induces the main effects of BDNF, as well as pro-BDNF, the molecule that after cleavage becomes the mature BDNF^67^.

Low-BDNF levels in newborns have also been associated with growth restriction and delivery for medical indications in the very preterm children^68^. This suggests that the lower BDNF levels seen in newborn ASD cases may be caused by less favorable pregnancy conditions.

BDNF correlated slightly negatively with CRP, while other inflammatory markers and neurotrophic factors correlated positively with BDNF (Supplementary Fig. 1). The highest correlation was seen between BDNF and VEGF-A. VEGF-A is a growth factor that stimulates vasculogenesis and angiogenesis after stress, and it is an essential factor for placental development^69^. VEGF-A plays an important role in the growth and differentiation of hematopoietic cells, which are the precursors to megakaryocytes that produce both blood platelets and BDNF^70^. Decreased blood levels of VEGF-A have also been described after clinical presentation of ASD, both in humans where VEGF-A levels were significantly lower in autism cases compared to Retts syndrome^71^, and in VPA-induced autism mouse models^72^. However, this is not corroborated in another study in humans^73^. Genetic polymorphisms leading to higher levels of circulating VEGF-A have been associated with larger gray and white matter brain volume and a higher cerebral blood flow^74^. Transplantation of VEGF-A producing cells has been shown to reduce neonatal brain damage after hypoxia-ischemia in rats^75^. Low levels of VEGF-A at birth may indicate that angiogenesis and vasculogenesis are not sufficiently stimulated, leading to impaired development of the immature newborn brain. It is possible that the low VEGF-A-levels are the cause for the observed lower BDNF-levels.

Patients with mental and neurodegenerative disorders often have reduced BDNF blood (and brain) levels and frequently exhibit neuroinflammation^76^. We found a trend toward elevated levels of inflammatory markers and reduced levels of neurodevelopmental markers in ASD cases. To further study this trend, we analyzed the pairwise correlation of BDNF with all other markers finding a positive correlation of BDNF with TARC and IgA and a negative correlation with CRP. Since all correlations were marginal, and no significant difference was found between cases and controls, our data cannot contribute in explaining the role of inflammation in the development of ASD.

It is known that ASD has genetic predispositions^18^, but we could not find them in the coding regions for BDNF. Haploinsufficiency of BDNF, present in human WAGR/11p13 deletion syndrome^77^, is associated with ASD, reduced adaptive behavior and cognitive functioning, social impairment, and higher score in the autism diagnostic test ADI-R. In the present study it was not possible to correlate newborn BDNF blood levels with the trajectory of ASD. However, the BDNF level did not correlate with the age at diagnosis. ASD subgroups—particularly those with regression of cognitive and social functions vs. the ASD patients without such regression—have been shown to differ with respect to biomarker profiles^78^. This would be an important subject of further study.

It appears that findings of increased or decreased blood levels of brain development factors associated with ASD are dependent on the time of blood sampling. That is, if the samples are drawn a few days after birth during a period of significant brain development and prior to recognizable symptoms, or if they are drawn later in life when the disease is obvious and debilitating, the relationship between cases and controls can change. Common for most studies are abnormal profiles of neuro-developmentally important factors in children with ASD, both before and after diagnosis. The fact that BDNF is decreased a few days after birth suggests that the neurodevelopmental change starts already in utero. The identification of biomarkers that could identify the younger siblings of ASD children that is of high risk for ASD might help in being able to start early treatment^79^. This is a major focus for ASD research.

The clear demonstration of reduced BDNF in blood in newborn children that develop ASD defines the BDNF associated pathways as a very promising area for further research^80^. It may also help identifying a long-sought marker for high ASD risk in, e.g., younger siblings of ASD children.

We could not find any significant neonatal biomarkers for schizophrenia, ADHD, bipolar- or affective disorders. More clear-cut cases with only one diagnose would be optimal, as the biomarkers for one disorder may be hidden by biomarker noise from another disorder. Further, some of the controls may be cases that has just not been diagnosed yet. Especially for the disorders mainly diagnosed in adults, it is possible that the biological changes has not appeared yet a few days after birth.

### Assay performance and stability

We found the assay to be very stable with generally small analytical variations. Some of the analytes were significantly degraded after one freeze–thaw cycle, and the analyte concentrations were significantly decreased after more than 30 years of storage. The filter paper was changed in 1989 from “Schleicher & Schuell grade 2992” to “Schleicher & Schuell grade 903”, which can explain some of the lower concentrations measured in the samples from 1980s, as it is known that there is a 17% difference in blood absorption between the two different papers^81^. In 2010 the paper was changed from “Schleicher & Schuell 903” to “Ahlstrom grade 226”, but these two paper qualities have been shown to absorb blood equally well^82^. In this study, we have therefore matched cases and controls on birth year, and all samples were treated identically with the same number of freeze–thaw cycles and the same number of days stored after extraction. In this way, we were able to both accommodate the different papers used and the degradation of analytes over the years. Further, all analyte concentrations were stratified by year using *z*-scores in the statistical calculations, to avoid that analyte degradation could have any influence on the statistical results.

### Final remarks

The strengths of this study are the large sample size and the homogeneity of the samples since all samples were drawn from the newborns at the same time after birth. The sample matrix has no variation since the samples are dried on the same kind of filter paper at room temperature. Further strengths are that the Danish Newborn Screening Biobank is population based, and this study includes samples from a broad cross section of society regardless of social and economic status. A weakness is that we only have one sample per person, thus we are only able to look at biomarkers at one time window. This means that we cannot see all potential neonatal biomarkers as some may already have disappeared and others have not yet appeared. For most markers, we do not know if the origin is fetal or maternal. There was some overlap in diagnoses between cases and controls, but not more than in a background population. These overlaps may reduce or twist the significance of the found biomarkers. Some cases had more than one diagnosis, thus the biomarkers for one disorder may be hidden by biomarkers for another disorder.

We found decreased blood levels of BDNF, an important factor for the developing brain, in blood from newborns later diagnosed with ASD. This encourages further research in the role of neurotrophic factors for the etiology of ASD.

## Supplementary information

Assay characteristics for protein measurements

Preanalytical variation

Correlation of BDNF-levels with other markers in ASD-cases

Forest plot for ADHD

Forest plot for schizophrenia

Forest plot for bipolar disorders

Forest plot for affective disorders

Concentrations of all biomarkers in cases and controls for the different disorders

## Data Availability

The statistical codes used for the calculations can be found at https://github.com/HagenC/BDNF.
