# Supplementary material for: Reduced neonatal brain-derived neurotrophic factor is associated with autism spectrum disorders
Source: Transl Psychiatry. 2019 Oct 7;9:252. doi: 10.1038/s41398-019-0587-2 (PMC6779749; doi:10.1038/s41398-019-0587-2)
Supplement: Supplementary file 1 — Assay characteristics for protein measurements [file 41398_2019_587_MOESM1_ESM.docx]

**Supplementary table 1. Assay characteristics for protein measurements**

|  | intraassay CV % | interassay CV % | Mean conc. |  |
| --- | --- | --- | --- | --- |
|  | n=40 | n=131 | DBSS | LOD |
| BDNF pg/ml | 12.7 | 26 | 762.2 | 13.6 |
| CRP µg/ml | 2.4 | 12.8 | 0.63 | 0.0001 |
| IL-18 pg/ml | 3.2 | 10.2 | 1655.3 | 5.7 |
| IL-8 pg/ml | 2.5 | 11.1 | 145.9 | 7.9 |
| IgA µg/ml | 7.1 | 60.5 | 3.14 | 0.015 |
| MCP-1 pg/ml | 3.4 | 9.7 | 2223.1 | 12.1 |
| S100B pg/ml | 2.8 | 13.8 | 3698.6 | 236 |
| TARC pg/ml | 4.0 | 15.3 | 6208.4 | 35.8 |
| VEGF pg/ml | 2.6 | 10.2 | 586.1 | 19.4 |
| mean | 3.3 | 13.3 |  |  |
|  |  |  |  |  |
