# Supplementary material for: Reduced neonatal brain-derived neurotrophic factor is associated with autism spectrum disorders
Source: Transl Psychiatry. 2019 Oct 7;9:252. doi: 10.1038/s41398-019-0587-2 (PMC6779749; doi:10.1038/s41398-019-0587-2)
Supplement: Supplementary file 2 — Preanalytical variation [file 41398_2019_587_MOESM2_ESM.docx]

**Supplementary table 2. Preanalytical variation**

|  | **year of sampling** | | | | | | | | | | **p-value** |
| --- | --- | --- | --- | --- | --- | --- | --- | --- | --- | --- | --- |
| **analyte** | **1982** | **1982**  **fr-th** | **1990** | **1990**  **fr-th** | **2000** | **2000**  **fr-th** | **2010** | **2010**  **fr-th** | **2016** | **2016**  **fr-th** | **freeze-thaw** |
| **BDNF pg/ml** | 148.6*** | 114.7^#^ | 733 | 561 | 862.2 | 687 | 1069 | 802.6 | 975.8 | 843.6 | **0.0246** |
| **CRP µg/ml** | 0.13*** | 0.10 | 0.33*** | 0.31 | 0.19*** | 0.19 | 1.89* | 1.94 | 4.16 | 3.57 | 0.9451 |
| **IL-18 pg/ml** | 1477* | 894^##^ | 3243** | 2428# | 2240 | 1918 | 2790 | 2814 | 2338 | 2056 | **0.0046** |
| **IL-8 pg/ml** | 62.1*** | 55.8 | 143.5** | 113.9 | 116.2*** | 108.9 | 291.9 | 258 | 357.9 | 286 | 0.6892 |
| **IgA µg/ml** | 0.69*** | 0.67 | 2.71 | 2.43 | 2.38* | 2.05 | 2.75* | 2.59 | 3.71 | 3.15 | 0.4549 |
| **MCP-1 pg/ml** | 1308*** | 980.1^#^ | 2407*** | 2258 | 2524*** | 2399 | 4139 | 4109 | 4278 | 4273 | 0.3974 |
| **S100B pg/ml** | 3364 | 1213^###^ | 8060 | 4469 | 5580 | 4692 | 4827 | 2954^#^ | 5521 | 3211 | **0.0003** |
| **TARC pg/ml** | 1883*** | 1413 | 6462* | 6156 | 3984*** | 4215 | 8297 | 8148 | 9174 | 8739 | 0.8422 |
| **VEGF pg/ml** | 364.3** | 216.1^###^ | 779.3*** | 554.3^#^ | 751.1** | 539.6 | 1212 | 829.8 | 1541 | 1155 | **0.0021** |
