# Supplementary material for: Reduced neonatal brain-derived neurotrophic factor is associated with autism spectrum disorders
Source: Transl Psychiatry. 2019 Oct 7;9:252. doi: 10.1038/s41398-019-0587-2 (PMC6779749; doi:10.1038/s41398-019-0587-2)
Supplement: Supplementary file 3 — Correlation of BDNF-levels with other markers in ASD-cases [file 41398_2019_587_MOESM3_ESM.docx]

**Supplementary figure 1. Correlation of BDNF-levels with other markers in ASD-cases**


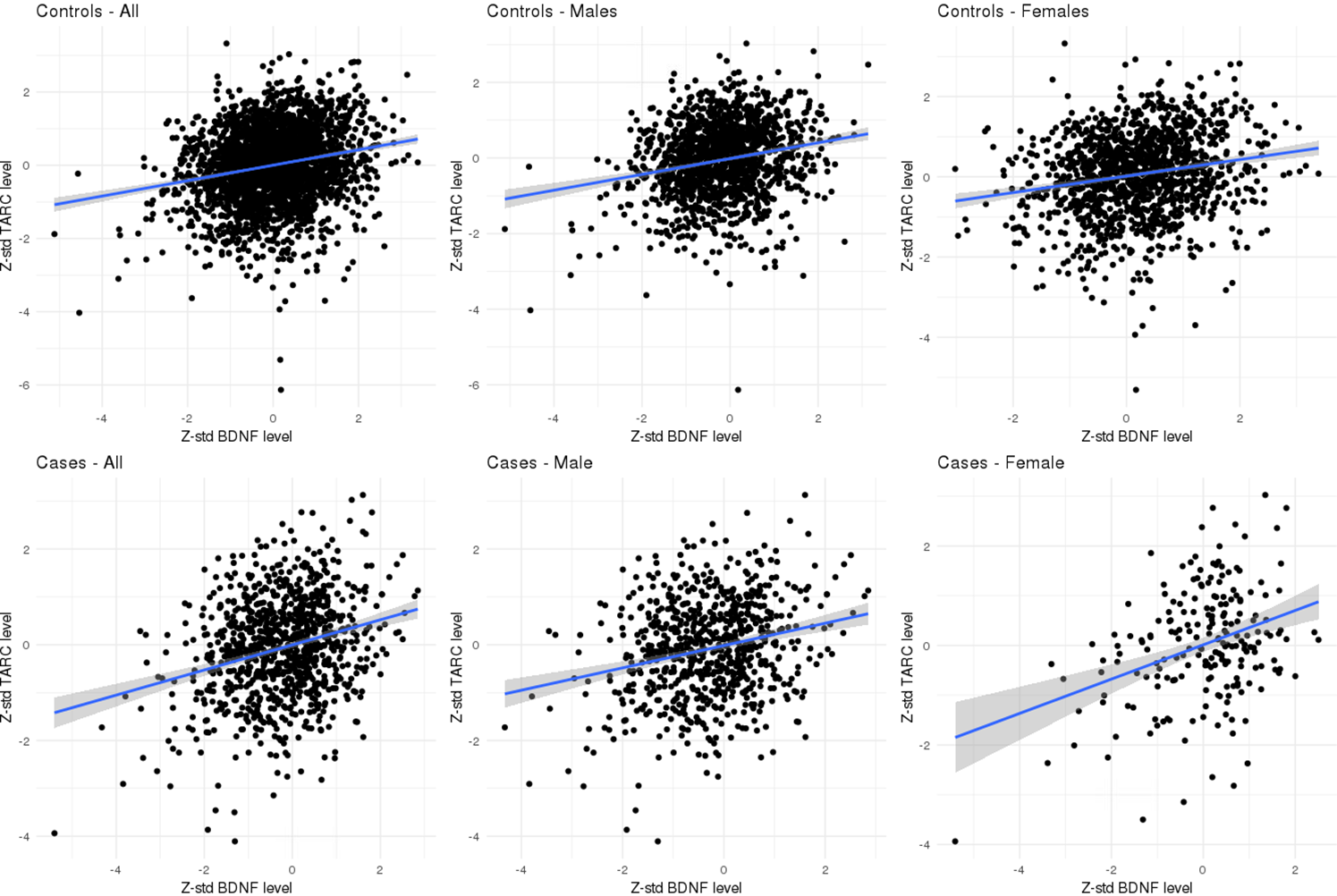

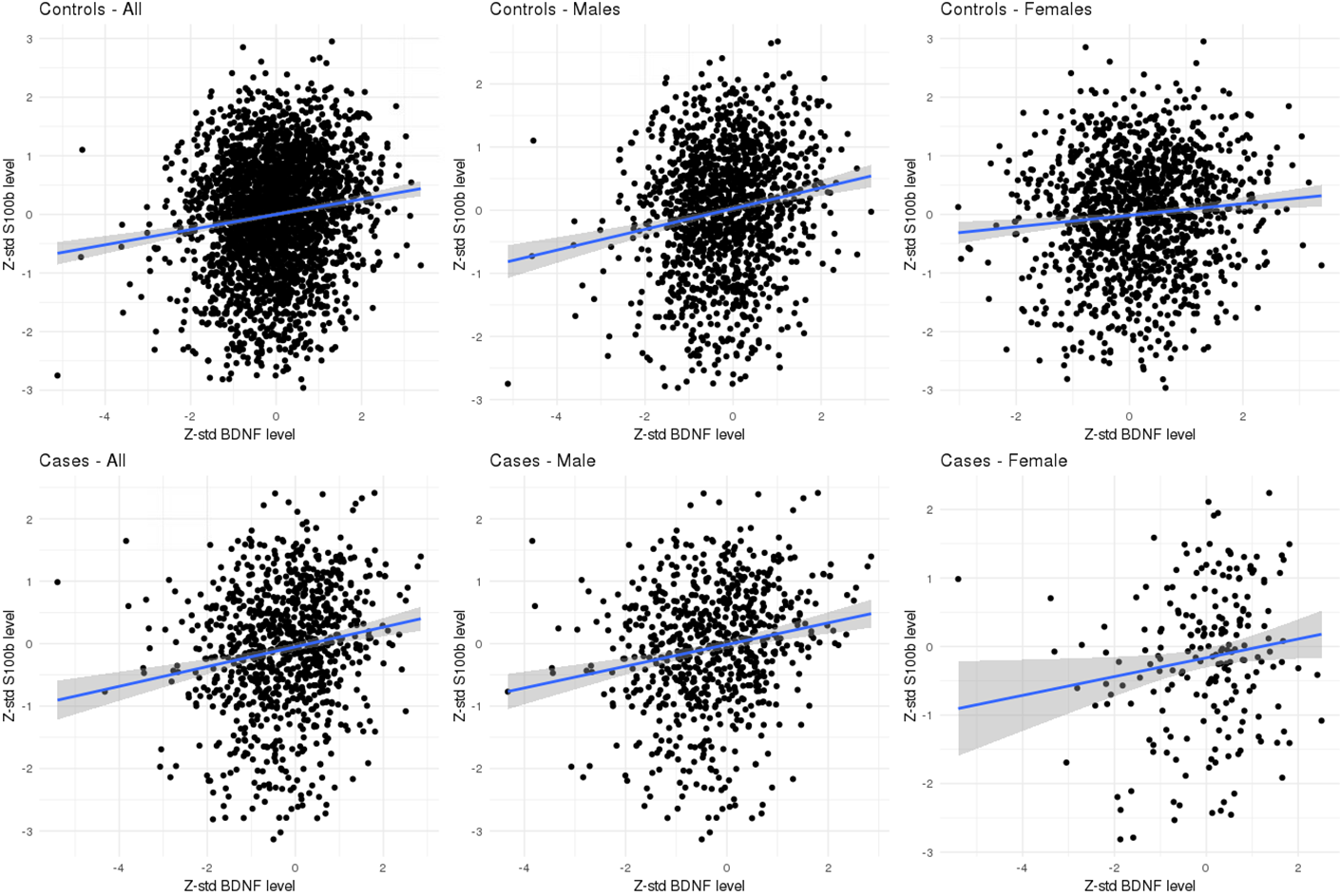

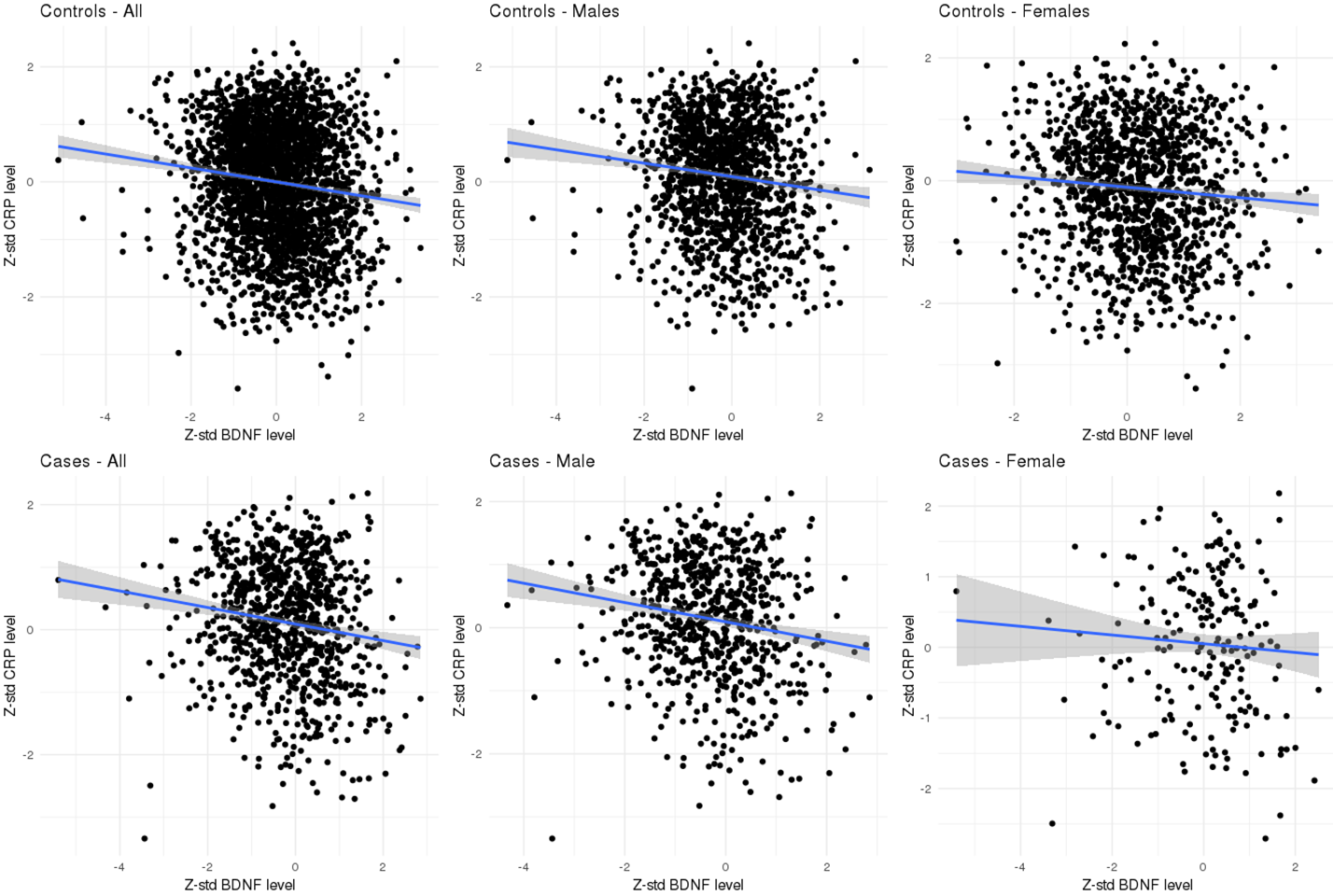


R^2^=0.022

P=4.75e^-06^

R^2^=0.032

P=2.62e^-08^

R^2^=0.043

P=1.38e^-10^


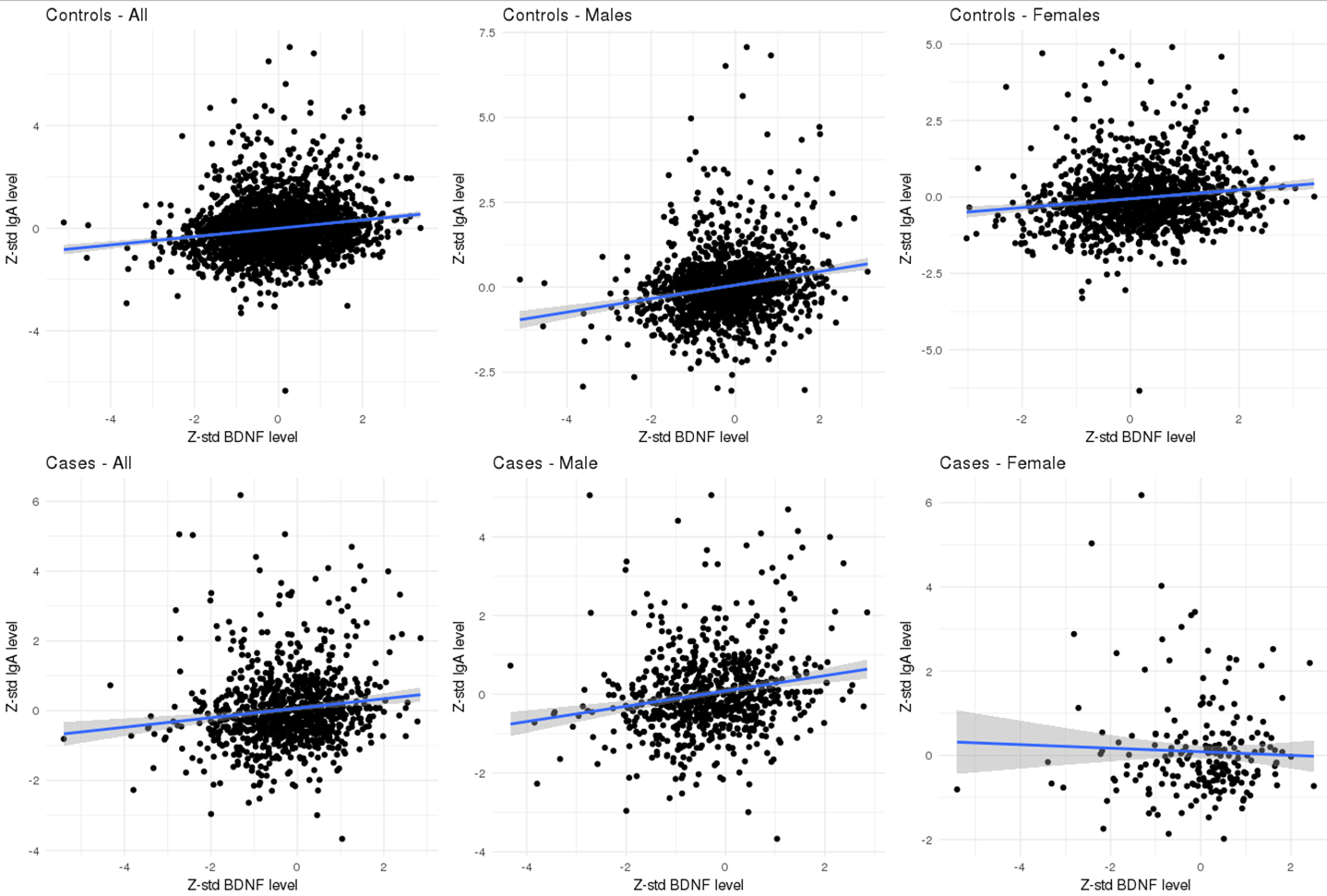

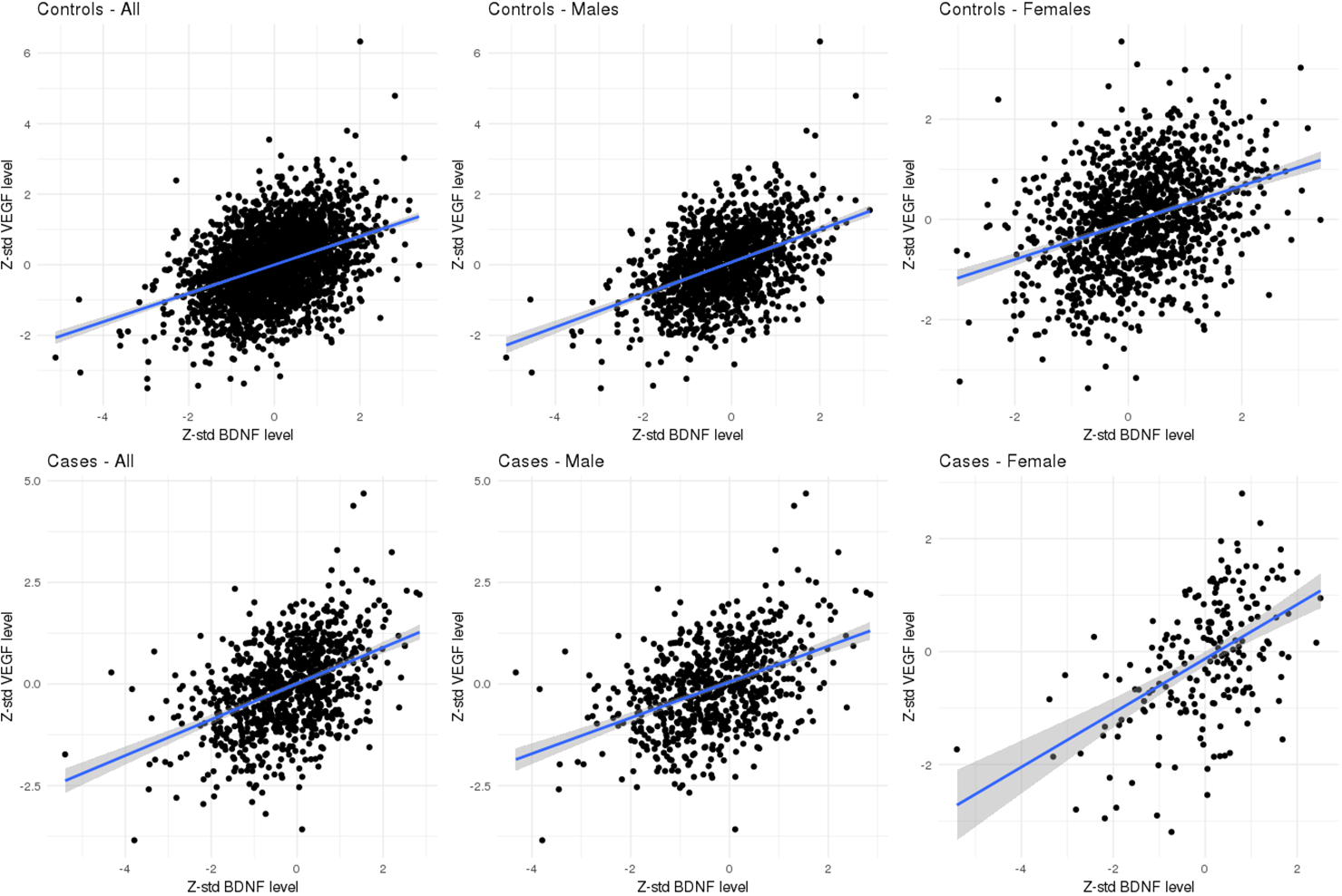


R^2^=0.025

P=8.74e^-07^

R^2^=0.177

P<2.0e^-16^
