# Supplementary figures and images for: Reduced neonatal brain-derived neurotrophic factor is associated with autism spectrum disorders
Source: Transl Psychiatry. 2019 Oct 7;9:252. doi: 10.1038/s41398-019-0587-2 (PMC6779749; doi:10.1038/s41398-019-0587-2)

**Supplementary figure 2. Forest plot for ADHD**


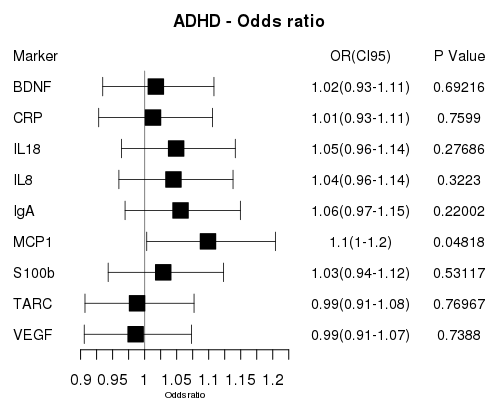

Supplement: Supplementary file 4 — Forest plot for ADHD [file 41398_2019_587_MOESM4_ESM.docx]

**Supplementary figure 3. Forest plot for schizophrenia**


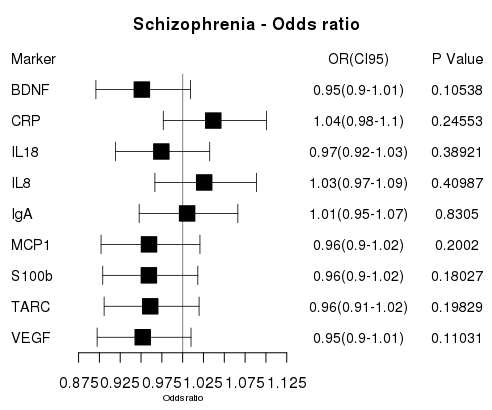

Supplement: Supplementary file 5 — Forest plot for schizophrenia [file 41398_2019_587_MOESM5_ESM.docx]

**Supplementary figure 4. Forest plot for bipolar disorders**


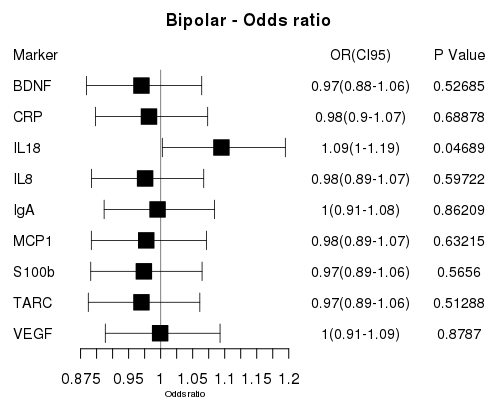

Supplement: Supplementary file 6 — Forest plot for bipolar disorders [file 41398_2019_587_MOESM6_ESM.docx]

**Supplementary figure 5. Forest plot for affective disorders**


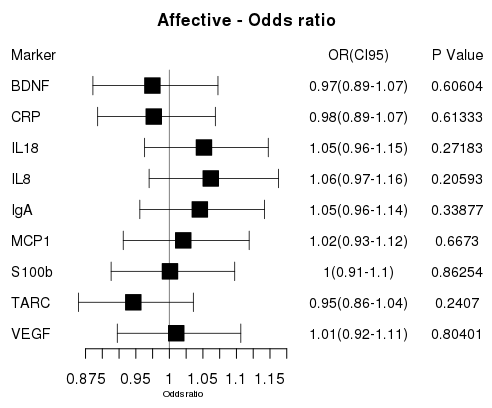

Supplement: Supplementary file 7 — Forest plot for affective disorders [file 41398_2019_587_MOESM7_ESM.docx]
