# Supplementary material for: Reduced neonatal brain-derived neurotrophic factor is associated with autism spectrum disorders
Source: Transl Psychiatry. 2019 Oct 7;9:252. doi: 10.1038/s41398-019-0587-2 (PMC6779749; doi:10.1038/s41398-019-0587-2)

**Supplementary figure 6. Concentrations of all biomarkers in cases and controls for the different disorders**

**a) BDNF**


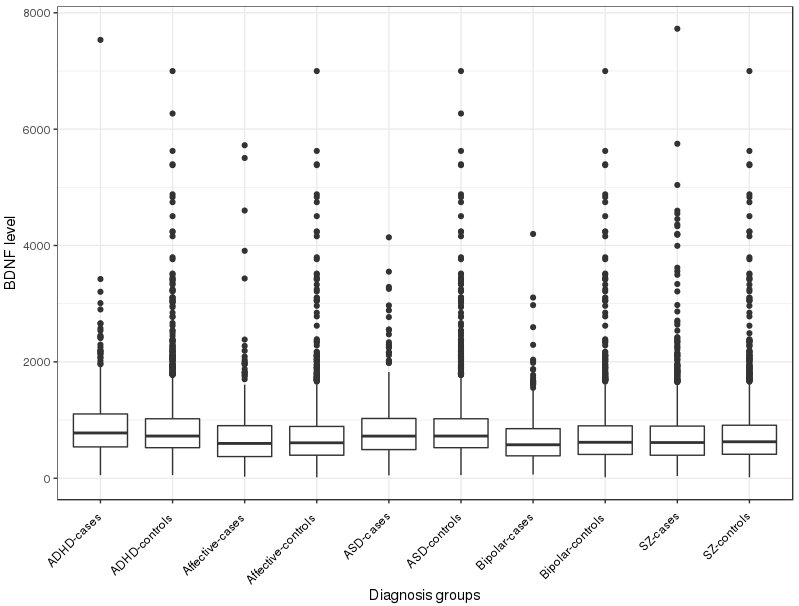


**b) CRP**


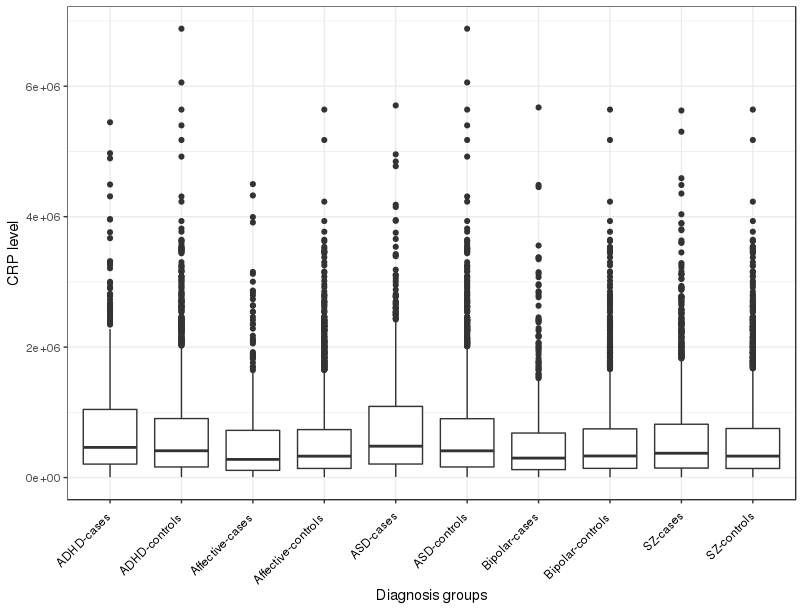


**c) IL-18**


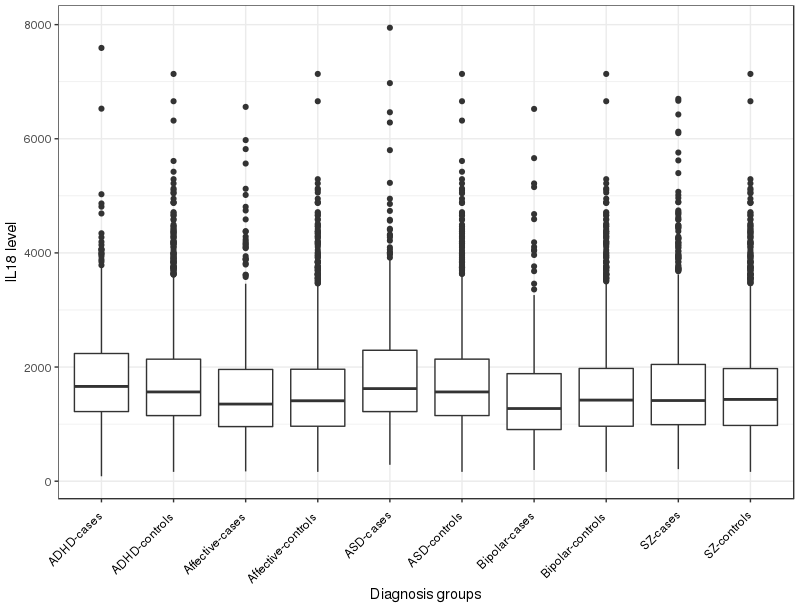


**d) IL-8**


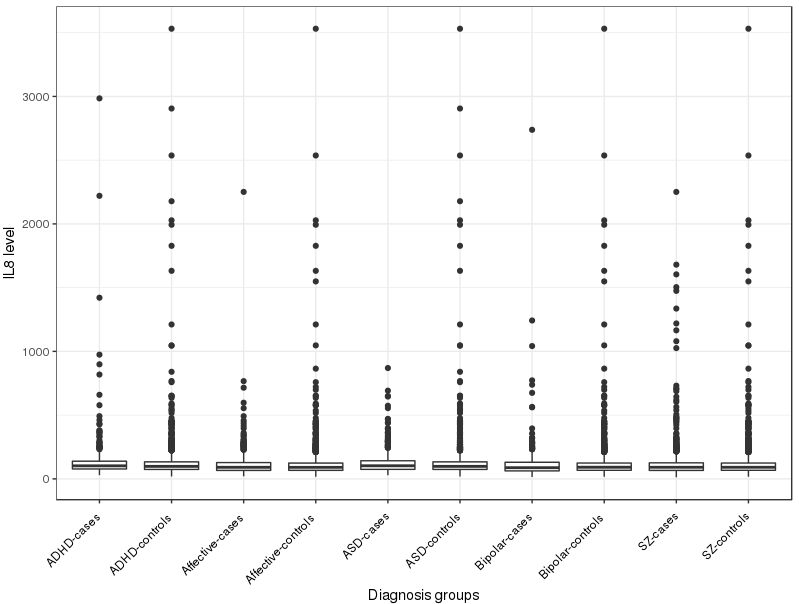


**e) IgA**


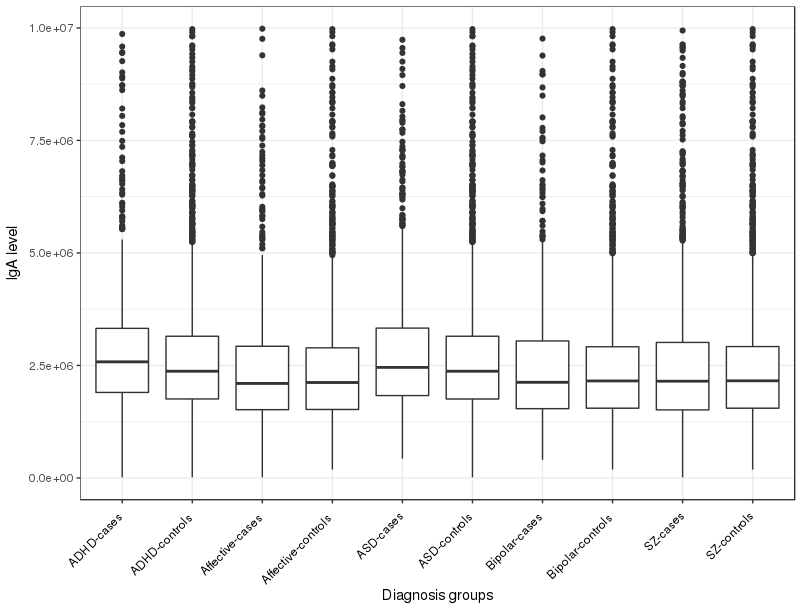


**f) S100b**


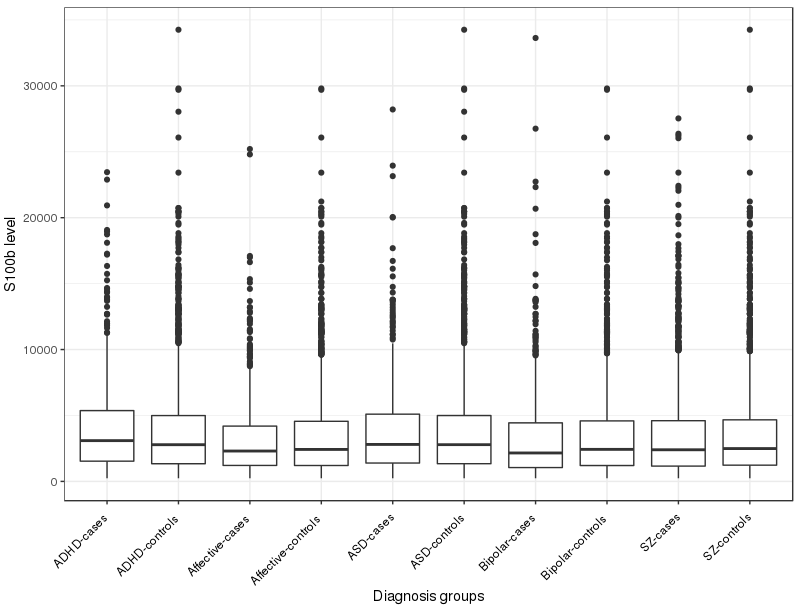


**g) TARC**


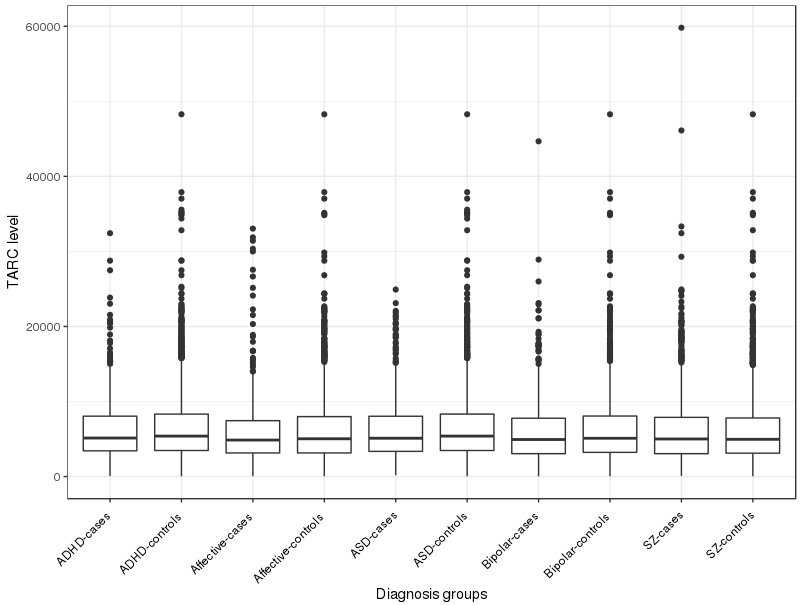


**h) VEGF-A**


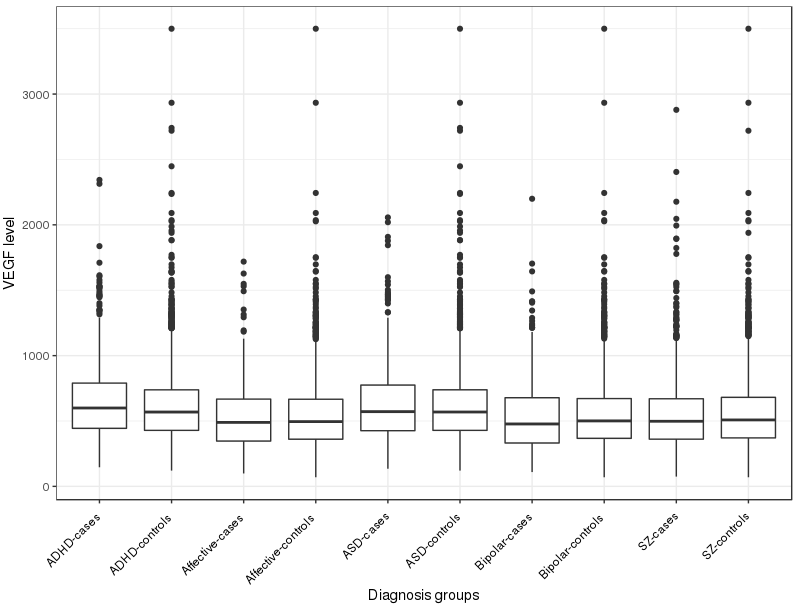


**i) MCP-1**


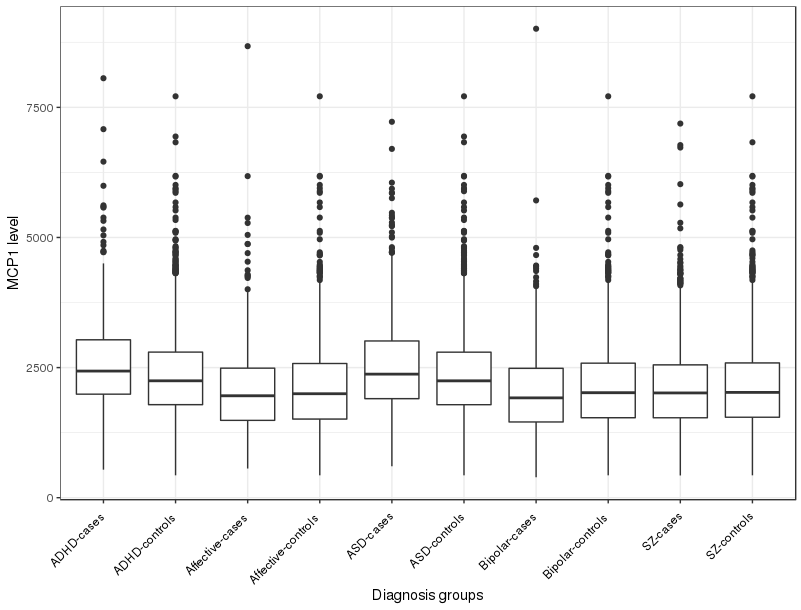

Supplement: Supplementary file 8 — Concentrations of all biomarkers in cases and controls for the different disorders [file 41398_2019_587_MOESM8_ESM.docx]
